# Supplementary material for: Facile Production of a Fenton-Like Photocatalyst by Two-Step Calcination with a Broad pH Adaptability
Source: Nanomaterials (Basel). 2020 Apr 3;10(4):676. doi: 10.3390/nano10040676 (PMC7221947; doi:10.3390/nano10040676)
Supplement: Supplementary file 1 [file nanomaterials-10-00676-s001.pdf]

Supplementary

Facile Production of a Fenton-Like Photocatalyst by Two-Step Calcination with a Broad pH Adaptability

Siyang Ji, Yanling Yang, Xing Li, Hang Liu and Zhiwei Zhou *

College of Architecture and Civil engineering, Beijing University of Technology, No.100 Xi Da Wang Road, Chao Yang District, Beijing 100124, China; jsy_3021826@163.com (S.J.); yangyanling@bjut.edu.cn (Y.Y.); lixing@bjut.edu.cn (X.L.); liuhang98596@163.com (H.L.)

* Correspondence: hubeizhouzhiwei@163.com; Tel.: +86-10-6739-1726

Received: 30 December 2019; Accepted: 31 March 2020; Published: date

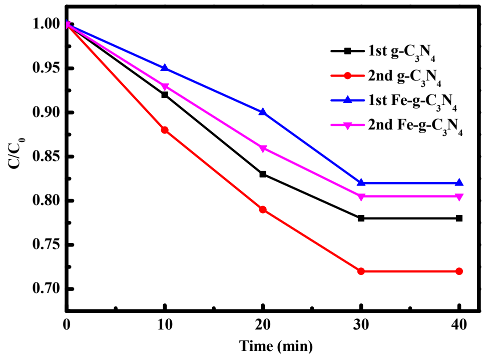

Figure S1. The dark adsorption of RhB by catalysts.

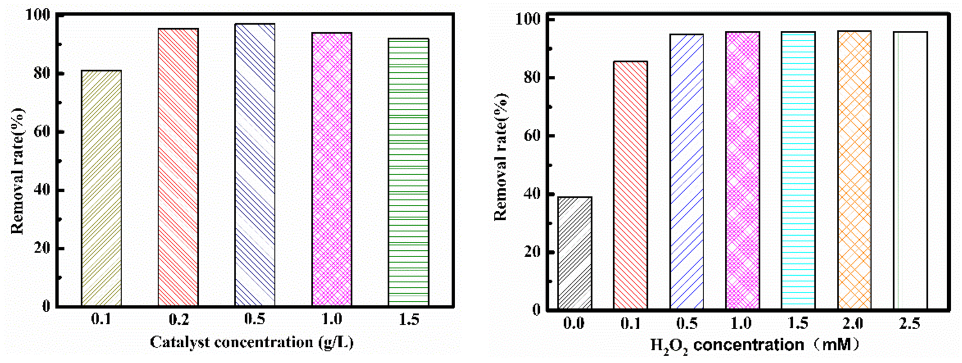

Figure S2. The effects of second Fe-g-C_3N_4 and H_2O_2 concentration on RhB degradation efficiency. The second Fe-g-C_3N_4 dosage was 20mg.

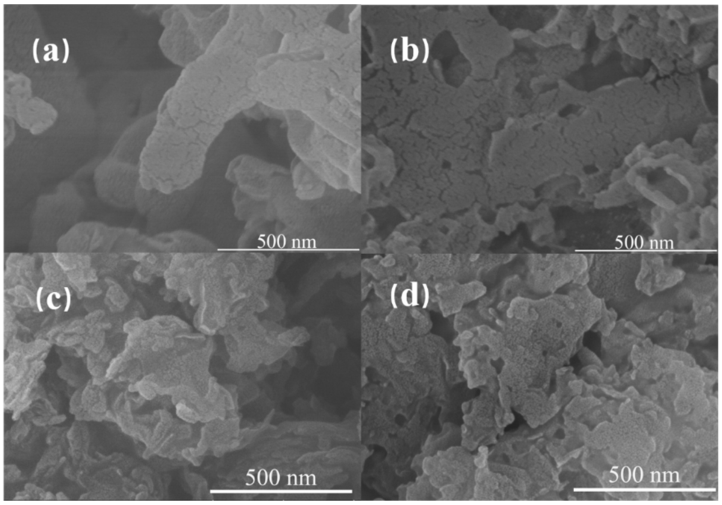

Figure S3. SEM micrograph of 1st-g-C_3N_4 (a), 1st-Fe-g-C_3N_4 (b), second g-C_3N_4(c), and 2nd Fe-g-C_3N_4 (d).

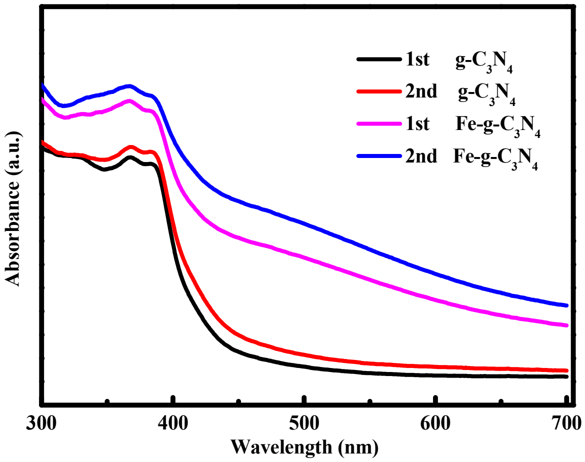

Figure S4. The UV-vis diffuse reflectance spectra of 1st-g-C_3N_4 , 1st-Fe-g-C_3N_4, 2nd g-C_3N_4 and second Fe-g-C_3N_4 .

Table S1. Structure parameters of the four samples.

Sample	Specific Surface area (m^2/g)	Pore diameter (nm)	Pore volume (cc/g)
1st-g-C_3N_4	56.779	15.827	0.243
2nd g-C_3N_4	78.535	21.601	0.388
1st-Fe-g-C_3N_4	45.605	16.553	0.162
2nd Fe-g-C_3N_4	63.521	22.592	0.248

Table S2. Atomic content (at %) from XPS analysis for samples.

Samples	C	N	O	Fe
1st-g-C_3N_4	42.43	53.81	3.75	
2nd-g-C_3N_4	43.84	51.59	4.59	
1st-Fe-g-C_3N_4	43.13	50.92	4.71	1.24
2nd-Fe-g-C_3N_4	40.91	50.58	7.07	1.44
